# Genetic breakthroughs in the *Brassica napus*–*Sclerotinia sclerotiorum* interactions

**DOI:** 10.3389/fpls.2023.1276055

**Published:** 2023-11-23

**Authors:** Rong-Shi Chen, Ji-Yi Wang, Rehman Sarwar, Xiao-Li Tan

**Affiliations:** School of Life Sciences, Jiangsu University, Zhenjiang, China

**Keywords:** plant defense, transcriptome, resistance gene, pathogen effector, oxalic acid, gene editing

## Abstract

*Sclerotinia sclerotiorum* (Lib.) de Bary is a highly destructive fungal pathogen that seriously damages the yield and quality of *Brassica napus* worldwide. The complex interaction between the *B. napus* and *S. sclerotiorum* system has presented significant challenges in researching rapeseed defense strategies. Here, we focus on the infection process of *S. sclerotiorum*, the defense mechanisms of rapeseed, and recent research progress in this system. The response of rapeseed to *S. sclerotiorum* is multifaceted; this review aims to provide a theoretical basis for rapeseed defense strategies.

## Introduction

Rapeseed (*Brassica napus*) is an allopolyploidy (AACC) resulting from the natural hybridization of *Brassica rapa* (AA) and *Brassica oleracea* (CC) (Chalhoub et al., 2014). It is an important and widely grown oil crop rich in oil and protein content (Chen et al., 2015; Chew, 2020). Rapeseed can be classified into three types based on its growth requirements: annual spring type, biennial winter type, and semi-winter type (Wang et al., 2011). Winter types necessitate an extended period of low-temperature vernalization before flowering, while spring types do not require this phase. Semi-winter types exhibit limited cold tolerance and are suitable for regions with moderately cold winter temperatures (>0°C) (Wang et al., 2011; Leijten et al., 2018; Matar et al., 2021). Rapeseed, oil palm, and soybean are the three primary sources of edible plant oil. “Double-low” rapeseed oil, also known as canola oil quality (glucosinolates <30 μmol/g, erucic acid <2%), is rich in unsaturated fatty acids (oleic acid, linoleic acid, linolenic acid, etc.) and various nutrients (phenols, phytosterols, vitamins, etc.). Consequently, the market share of rapeseed oil is consistently on the rise (Beyzi et al., 2019; Ye and Liu, 2023). Rapeseed is also used in animal feed and biofuel production, with high economic value and market potential (Jiang et al., 2019).

*Sclerotinia sclerotiorum* (Lib.) de Bary is the ascomycetes’ highly destructive fungal pathogen. It is widespread and infects over 700 plant species, including many economically important crops like rapeseed, sunflower (*Helianthus annuus*), peanut (*Arachis hypogaea*), soybean (*Glycine max*), garden lettuce (*Lactuca sativa*), and sugar beet (*Beta vulgaris*) (Boland and Hall, 1994; Uloth et al., 2013; Xia et al., 2019; Zhang et al., 2021). Sclerotinia stem rot (SSR) is the primary disease caused by *S. sclerotiorum*, resulting in the necrosis of stems and leaves in rapeseed, which can lead to yield losses of up to 80% in severe cases (Li et al., 2006a). *S. sclerotiorum* exists in the form of resting bodies known as sclerotia, which can withstand harsh environmental conditions. When the environment becomes suitable for growth, these sclerotia germinate, adopting either a myceliogenic or carpogenic form, producing hyphae or apothecia. These apothecia release ascospores that infect plant tissue (Bolton et al., 2006). Ascospores land on plant tissues, germinate as mycelium, and secrete oxalic acid (OA), cell wall degrading enzymes (CWDEs), and other substances to facilitate colonization (Kabbage et al., 2015; Xia et al., 2019). Previous studies have shown the critical role of OA synthesis and secretion in the pathogenesis of *S. sclerotiorum*, with fungal mutants deficient in OA production being non-pathogenic. The pathogen elevates the level of reactive oxygen species (ROS), and it induces a hypersensitive response (HR) in plant cells by secreting OA into plant tissues, ultimately leading to programmed cell death (PCD) (Godoy et al., 1990; Kim et al., 2008). No varieties with complete resistance to SSR have been reported in rapeseed. Disease control primarily relies on field management and fungicide application. However, this requires growers to predict the timing of *S. sclerotiorum* infection, which may also result in environmental pollution and presents several limitations due to the lack of effective prediction methods (Bolton et al., 2006).

In recent years, the increasing global demand for rapeseed has led to intensified cultivation practices, increasing the urgency for effective strategies against *S. sclerotiorum*. Developments in various tools, techniques, and expanded research on *S. sclerotiorum* have generated valuable insights into defense strategies for rapeseed. Here, we introduce the infection process of *S. sclerotiorum*, the defense strategies of rapeseed, recent research on the identification of defense-related genes in rapeseed, and the resistance strategies of transgenic rapeseed. The *B. napus*–*S. sclerotiorum* system has a complex regulatory network, and this review aims to provide a reference for future studies on *S. sclerotiorum* resistance strategies in rapeseed.

## The infection process of *S. sclerotiorum*

**Figure 1:**
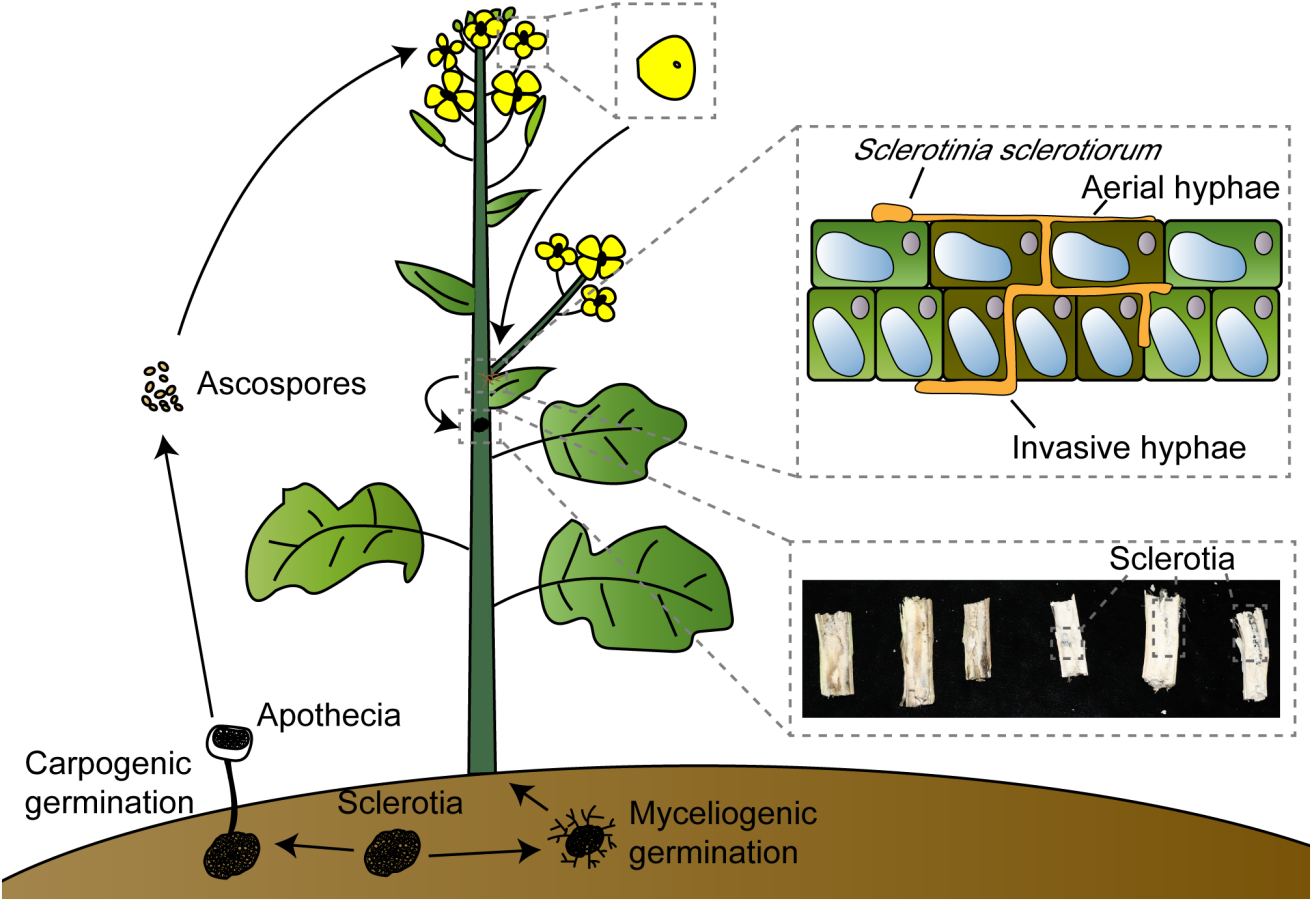
*Sclerotinia sclerotiorum* invasion model. *Sclerotinia sclerotiorum* can germinate under suitable conditions, myceliogenic germination produces hyphae to infect plant roots; carpogenic germination produces apothecia, and ascospores are released into the air by apothecia. Ascospores land on petals, which tend to gather in the leaf axils when they fall off. *Sclerotinia sclerotiorum* will colonize by aerial hyphae and invasive hyphae; invasive hyphae thicken after entering the apoplast and associate with early biotrophic growth, and finally produce sclerotia in the stem leading to hollow and rot.

*S. sclerotiorum* is a typical necrotrophic pathogen, and recent studies have revealed that it undergoes a transient biotrophic phase after initially colonizing plant tissues before transitioning into a necrotrophic pathogen (Kabbage et al., 2015; Liang and Rollins, 2018). The sclerotia present in the soil exhibit two states in relatively high-humidity conditions: myceliogenic germination, which produces hyphae to infect plant roots, and carpogenic germination, leading to the production of apothecia from which ascospores are released into the air (Clarkson et al., 2003; Hegedus and Rimmer, 2005). Typically, ascospores colonize senescent tissues, primarily petals, rather than healthy tissues. Under suitable conditions, they will generate hyphae (Turkington et al., 1991; Jamaux et al., 1995). Subsequently, infection cushions are formed to breach the host cuticle, entering a brief biotrophic phase in the apoplast. During this phase, OA and some hydrolytic enzymes are synthesized and secreted to inhibit the oxidative burst of host cells and host defense, degrade the cell wall, and the composite effect of infection cushions enhances penetration and expedites pathogen colonization. After penetrating the plant’s cuticle, *S. sclerotiorum* generates subcuticular vesicles and subcuticular infection hyphae that spread below the cuticle, indicating the beginning of the biotrophic phase, accumulate nutrients needed for hyphal growth, and serve as the foundation for colonization by *S. sclerotiorum*. The branches of subcuticular infection hyphae will produce ramifying hyphae, and they will penetrate anticlinal cell wall junctions, which will reduce the stability of the epidermal cell wall (Jamaux et al., 1995; Huang et al., 2008). Finally, the pathogen enters the necrotrophic phase (Garg et al., 2010; Kabbage et al., 2015; Derbyshire and Denton-Giles, 2016). As the petals fall, the hyphae spread to other healthy plant parts, such as leaves or stems, and may also extend to adjacent plants, causing stem rot and severe yield losses (**Figure 1**).

OA plays a multifaceted role in the pathogenesis of *S. sclerotiorum*. In the early stages of infection, OA accumulation in infected tissues significantly impacts the host’s redox environment. This effect leads to the suppression of the oxidative burst, the inhibition of callose deposition, and a reduction in ROS production. Consequently, it hampers the plant defense response. OA also contributes to the chelation of Ca^2+^, influencing Ca^2+^ signal transduction and pectin structure, and it leads to a decrease in environmental pH, enhancing the activity of CWDEs (Favaron et al., 2004; Williams et al., 2011; Fagundes-Nacarath et al., 2018). In the later stages of infection, the induction of ROS production leads to HR or PCD in host cells (Liang et al., 2009). OA-deficient mutants of *S. sclerotiorum* are nonpathogenic because they cannot alter the host’s redox environment; the strain cannot colonize but can induce strong HR-like plant defense (Williams et al., 2011). Most hosts of *S. sclerotiorum* are dicotyledons, which may be associated with OA preference (Derbyshire et al., 2022). Germin-like proteins (GLPs) were found in cereals with oxalate oxidase (OXO) activity and can break down OA into CO_2_ and H_2_O_2_ (Lane et al., 1993; Woo et al., 2000; Davidson et al., 2009). Rapeseed overexpressing the *OXO* gene showed higher tolerance to OA and stronger resistance to *S. sclerotiorum* (Zou et al., 2007; Dong et al., 2008; Liu et al., 2015). This may explain why *S. sclerotiorum* is unable to infect many monocotyledons.

Pathogens secrete effectors that play an essential role in the pathogenesis process, and these small secreted proteins can either promote or inhibit host cell death according to the pathogens (Hogenhout et al., 2009; Lyu et al., 2016). A previous study based on whole genome sequencing estimated the presence of approximately 70 putative effector genes in *S. sclerotiorum* (Derbyshire et al., 2017). Some effectors have been identified in *S. sclerotiorum*. PGIP-INactivating Effector 1 (SsPINE1) can inactivate plant polygalacturonase-inhibiting proteins (PGIPs), promoting the dissociation of polygalacturonases (PG)-PGIP and enhancing necrotrophic virulence (Wei et al., 2022). Necrosis and ethylene‐inducing peptides 1 and 2 (SsNep1, SsNep2) caused necrosis by transient expression in tobacco leaves (Dallal Bashi et al., 2010). Integrin alpha N-terminal domain superfamily member SsITL inhibits the expression of *PLANT DEFENSIN1.2* (*PDF1.2*) in *A. thaliana*, affecting the plant defense response (Zhu et al., 2013). A cutinase, SsCut, causes cell death in some host plants, including *B. napus*, *G. max*, and *Oryza sativa* (Zhang et al., 2014a). SsSSVP1 affects the plants’ energy metabolism and promotes infection (Lyu et al., 2016). Intracellular necrosis‐inducing effector 1 (SsINE1) can enter host cells by the RxLR‐like motif, and SsINE5 causes necrosis by nucleotide‐binding leucine‐rich repeat (NLR) proteins (Newman et al., 2023). Cerato-platanin protein 1 (SsCP1) can target plant PR1 and cause host cell death (Yang et al., 2018). YML079-like cupin protein (SsYCP1) can promote pathogen infection and play an essential role in the pathogenesis of *S. sclerotiorum* (Fan et al., 2021a). These effectors are crucial elements in the study of interactions between plants and *S. sclerotiorum*, providing insights into the mechanisms underlying the pathogenic process.

## Rapeseed defense response to *S. sclerotiorum* infection

Plant immunity against pathogens comprises two main branches: pattern-triggered immunity (PTI) and effector-triggered immunity (ETI). PTI relies on transmembrane pattern recognition receptors (PRRs) that recognize pathogen-associated molecular patterns (PAMPs), while ETI involves the use of proteins encoded by intracellular resistance (R) genes. These mechanisms contribute to plant defense together (Jones and Dangl, 2006). The involvement of the PTI has been demonstrated in plants–*S. sclerotiorum* system (Zhang et al., 2013). Several receptor-like protein (RLP) genes, including candidates such as *BnaA02g16770D* and *BnaC02g22760D*, have been identified in *B. napus* in response to *S. sclerotiorum* (Li et al., 2022). Various endogenous hormones regulate the downstream immune responses in plants against pathogens through a series of signal transduction pathways. Signal pathways mediated by salicylic acid (SA), jasmonic acid (JA), and ethylene (ET) play significant roles in regulating plant defense responses (Thomma et al., 2001; Pieterse et al., 2012). Studies in *Arabidopsis thaliana* have revealed that SA can induce PCD and HR reactions at the infection site, providing resistance to biotrophic pathogens. The JA/ET signaling pathway, independent of the SA pathway, confers resistance to necrotic pathogens (Glazebrook, 2005; Li et al., 2019). These signaling pathways have essential roles in the defense of rapeseed against *S. sclerotiorum*, and in particular, the SA pathway was shown to be involved in the defense response against necrotrophic pathogens (Nováková et al., 2014). SA and JA pathways often exhibit antagonistic interactions. Positive regulatory genes in the SA pathway, such as WRKY70, negatively regulate the JA pathway, while *MPK4* plays an opposite role. During *S. sclerotiorum* infection, SA and JA levels peak at 12 hours post-infection (hpi) and 24 hpi, respectively. The expression changes of SA–JA crosstalk genes such as *BnWRKY70*, *BnNPR1*, *BnMPK4*, and *BnEDS1* suggest the importance of the orderly expression of SA and JA pathways (Wang et al., 2012). Additionally, a study in *Brassica carinata* showed a higher tolerance to *S. sclerotiorum* infection compared to *B. napus*, suggesting that differences in the timing of SA and JA pathways may be the contributing factor while indicating the potential importance of the ET pathway in plant defense (Yang et al., 2010). The hormone-mediated complex defense network is essential to the plant immune system. While synergistic and antagonistic effects of SA, JA, and ET pathways are relatively well-understood, research on the ET pathway in the context of the *B. napus*–*S. sclerotiorum* system remains somewhat limited, warranting further exploration in future studies.

## *B. napus*–*S. sclerotiorum* system analysis

Transcriptome analysis of the *B. napus*–*S. sclerotiorum* interaction provides a comprehensive understanding of the molecular events during *S. sclerotiorum* infection and the defense strategies in rapeseed. Illumina sequencing was conducted at various time points following *S. sclerotiorum* infection, leading to the categorization of differentially expressed genes (DEGs) into several major groups, including hydrolytic enzymes, secondary metabolites, detoxification processes, signaling pathways, developmental processes, secreted effectors, OA regulation, and the production of ROS. The results revealed a noteworthy increase in the expression of genes related to nucleic acid binding during the brief biotrophic phase (12–24 hpi), coinciding with the upregulation of genes such as the pathogen-induced cutinase A gene *SsCuta* and lipid degradation-related genes *SS1G_09557*, *SS1G_01953*, and *SS1G_11930*; these lipolytic enzymes also seem to be involved in cuticle penetration facilitated by the mechanical pressure applied by infection cushions during the same period (Bashi et al., 2012). Additionally, genes associated with biotrophic interactions exhibited heightened expression during this phase (Lumsden and Wergin, 1980). Plant tissue necrosis typically occurs at 24 hpi, indicating the beginning of the necrotic phase; during this stage, the expression of multiple genes encoding hydrolases and related to secondary metabolite syntheses and toxin increases, such as nonaspartyl acid protease (*SsACP1*), polyketide synthase (*SsPKS*), and non-ribosomal peptide synthase (*SsNRPS*), and genes encoding subtilisin-like serine proteases (*SS1G_07655, SS1G_02423*, and *SS1G_032820*) can degrade cell wall glycoproteins and may play an essential role in fungal customization (Olivieri et al., 2002). In late infection, the expression of genes encoding PGs, cellulases, β-1,4-glucanases, arabinogalactan-degrading enzymes, mannosidases, laccases, and necrosis and ET-inducing peptides increased (Dallal Bashi et al., 2010). Genes such as *SsOAH* encoding oxaloacetate acetylhydrolase showed consistent expression during infection. Still, they exhibited heightened expression in the middle and late stages, paralleling similar trends in the gene encoding oxalate decarboxylase. The dynamic accumulation of OA seems to play an important role in pathogen infection. Detoxification is essential for pathogen resistance against host defenses, involving the modification or degradation of host-produced antitoxins or their removal from host cells. Genes encoding cytochrome P450 enzymes (*SS1G_02340*) and eburicol 14 alpha-demethylase (*CYP51* and *SS1G_04805*) were upregulated in the early stages of infection (Seifbarghi et al., 2017). The expression of multiple genes encoding membrane transporters, such as significant facilitator superfamily (MFS) and ATP-binding cassette (ABC) transporters, increased in the late stage of infection. Some genes encoding cyanide hydratases/cyanate hydrolases and 2-nitropropane dioxygenases exhibited varying levels of upregulation at different stages of infection (Seifbarghi et al., 2017; Xu et al., 2021). Furthermore, Brassicales plants can produce chemicals for defense, such as isothiocyanates (ITCs), nitriles, and flavonols (Wittstock and Gershenzon, 2002; Cartea et al., 2010). ITCs and nitrile are produced from glucosinolates (GLs) via β-thioglucoside glucohydrolase enzymes (myrosinases) (Rask et al., 2000). Flavonols, including quercetin, kaempferol, isorhamnetin, and other polyphenolic compounds, are also part of this defense system (Cartea et al., 2010). Chen et al. (2019); Chen et al. (2020) identified two detoxification-related genes in *S. sclerotiorum*, *SsQDO* (quercetin dioxygenase gene), and *Ss12040* (ITC hydrolases gene). *SsQDO* deletion lines showed decreased pathogenicity and increased sensitivity to flavonols. *Ss12040*, a homolog of *SaxA* from *Pseudomonas syringae*, was essential for pathogenicity on ITC-defended plants. Together, these genes constitute the defense network of *S. sclerotiorum* against the antitoxins produced by rapeseed. Furthermore, salicylate hydroxylase and specific extracellular effectors, such as SsLysM and SsPINE1, are very important in inhibiting plant defense pathways (Ambrose et al., 2015; Peng et al., 2017; Seifbarghi et al., 2017; Xu et al., 2021). **Table 1** contains a list of pathogenicity-related genes in *S. sclerotiorum*.

**Table 1:** Pathogenicity-related genes of *S. sclerotiorum*.

Gene	Protein	Function	Transgenic method	Pathogenicity	Citations
*SsAGM1*	*N*-acetylglucosamine-phosphate mutase	Involved in growth, development, and pathogenicity	RNAi	Reduce	(Zhang et al., 2022a)
*Ssams2*	GATA-type transcription factor	Involved in chromosome segregation and cell division	RNAi	Reduce	(Liu et al., 2018b)
*SsBi1*	Putative BAX inhibitor-1 protein	Involved in pathogenesis and stress response	RNAi	Reduce	(Qu et al., 2014)
*SsCak1*	CDK-activating kinase	Essential for both growth and pathogenicity regulation	HIGS	Reduce	(Qin et al., 2023)
*SsCat2*	Catalase	Regulate oxidative stress	Knockout	Reduce	(Huang et al., 2021)
*Sscnd1*	Magnaporthe appressoria-specific (MAS) protein homolog	Involved in hyphal growth and compound appressorium formation	RNAi/host‐induced gene silencing (HIGS)	Reduce	(Ding et al., 2021b)
*SsCox17*	Mitochondrial copper metallochaperone	Transport copper ions to cytochrome c oxidase	RNAi	Reduce	(Ding et al., 2022)
*SsCP1*	Cerato-platanin protein	Target plant PR1	Knockout	Reduce	(Yang et al., 2018)
*SsCut1*	Cutinase	Break down plant cuticles	Knockout	Reduce	(Gong et al., 2022)
*SsEmp24/SsErv25*	p24 family protein	Involved in modulating morphogenesis and pathogenicity	Knockout	Reduce	(Xie et al., 2021)
*SsERP1*	Ethylene pathway repressor protein	Inhibit plant ethylene signaling pathway	RNAi	Reduce	(Fan et al., 2021b)
*SsFkh1*	Forkhead-box (FOX)-containing protein	Regulate sclerotium and compound appressorium development	Knockout	Reduce	(Cong et al., 2022)
*SsFoxE3*	Forkhead‐box family transcription factors	Involved in compound appressorium formation	Knockout	Reduce	(Jiao et al., 2022)
*SsITL*	Integrin alpha N-terminal domain superfamily	Inhibit plant defense	RNAi	Reduce	(Zhu et al., 2013)
*SsMADS*	MADS-box transcription factor	Involved in fungal growth and disease	RNAi	Reduce	(Qu et al., 2014)
*SsNEP2*	Necrosis and ethylene-inducible peptide	Induce ROS production in plant cells	Knockout	Reduce	(Yang et al., 2022)
*SsOah*	Oxaloacetate acetylhydrolase	Involved in OA accumulation, pH‐responsive growth, morphogenesis, and virulence	Knockout	Reduce	(Liang et al., 2015a)
*Ssodc2*	Oxalate decarboxylase	Involved in compound appressorium formation and function	Knockout	Reduce	(Liang et al., 2015b)
*Sspac1*	Zinc finger transcription factor	Involved in sclerotial development and virulence	Knockout	Reduce	(Rollins, 2003)
*SsPemG1*	Elicitor-homologous protein	Associated with infection cushions	RNAi	Enhance	(Pan et al., 2015)
*SsQDO*	Quercetin dioxygenase	Cleavage of the flavonol carbon skeleton	Knockout	Reduce	(Chen et al., 2019)
*SsRhs1*	Rhs repeat‐containing protein	Play an important role in virulence	RNAi	Reduce	(Yu et al., 2017)
*SsSaxA*	ITCase	Degrade ITCs	Knockout	Reduce	(Chen et al., 2020)
*SsShk1*	Histidine kinase	Control stress response, sclerotial formation, and fungicide resistance	Knockout	–	(Duan et al., 2013)
*SsSSVP1*	Secreted protein	Manipulate plant energy metabolism and promote infection	RNAi	Reduce	(Lyu et al., 2016)
*SsSte12*	Transcription factor	Involved in vegetative mycelial growth, sclerotia development, appressoria formation, and penetration-dependent pathogenicity	RNAi	Reduce	(Xu et al., 2018)
*SsSvf1*	Survival factor	Response to oxidative stress and involved in appressoria formation	RNAi	Reduce	(Yu et al., 2019)
*SsTOR*	Ser/Thr protein kinase	Regulate cell growth and metabolism	Knockout	Reduce	(Jiao et al., 2023)
*SsTrx1*	Thioredoxin	Involved in *S. sclerotiorum* development, oxidative stress	HIGS	Reduce	(Rana et al., 2021)
*SsXyl1*	Endo-β-1, 4-xylanase	Involved in growth and virulence	Knockout	Reduce	(Yu et al., 2016)
*SsYCP1*	YML079-like cupin protein	Promote infection	RNAi	Reduce	(Fan et al., 2021a)

Transcriptome analysis revealed the activation of the mitogen-activated protein kinase (MAPK) signaling pathway during the early stages of *S. sclerotiorum* infection. Multiple *MAPKKK*, *MKK*, and *MPK* genes were induced by *S. sclerotiorum*. The response of transcription factors (TFs) was intricate, with most TFs in families such as WRKY, MYB, NAC, and others being downregulated. The critical gene *ICS1* in SA biosynthesis showed an initial increase in expression followed by a decrease during the infection period (Zheng et al., 2015). Conversely, critical genes in JA biosynthesis, *AOS*, and *LOX2* exhibited increased expression over time (Wasternack, 2007). However, the accumulation of JA could not impede the infection’s progression due to the downregulation of *COI1*. This downregulation led to an increased abundance of the jasmonate zinc finger inflorescence meristem (ZIM) domain protein (JAZ) (Chini et al., 2007; Ruan et al., 2019). JAZ inhibited the JA signaling pathway and caused the downregulation of *MYC2*, preventing the activation of downstream genes of JA-related genes (Kazan and Manners, 2013). The ET pathway regulates plant growth development and participates in plant stress response. ETHYLENE RESPONSE FACTOR 1 (ERF1) and OCTADECANOID-RESPONSIVE ARABIDOPSIS ETHYLENE/ERF 59 (ORA59) are considered to be key signaling molecules in the JA-ET pathway (van Loon et al., 2006; Pré et al., 2008). Following *S. sclerotiorum* infection, the expression of *ETHYLENE RESPONSE SENSOR* (*ERS*), *ERF*, and *ORA59* increased, and the activation of the JA/ET pathway resulted in elevated expression of genes responsible for chitinase and pathogenesis-related (PR) genes such as *PDF1.2*, *PR2*, *PR3*, and *PR4*. This underlines the significance of the JA/ET pathway in defense against *S. sclerotiorum.* Additionally, the upregulation of *PGIPs* serves to inhibit PG in the cell wall, preventing cell wall degradation (De Lorenzo and Ferrari, 2002; Joshi et al., 2016b; Wei et al., 2016; Wu et al., 2016; Girard et al., 2017; Jian et al., 2018; Xu et al., 2021). Long non-coding RNAs (lncRNAs) and microRNAs (miRNAs) are essential in regulating the expression of plant genes in response to stress. While the expression of various lncRNAs and miRNAs changes following *S. sclerotiorum* infection, further research is needed to elucidate their precise roles in the defense response of rapeseed (Cao et al., 2016; Joshi et al., 2016a; Jian et al., 2018).

## Defense-related genes in rapeseed

Identifying essential defense genes in rapeseed is a necessary strategy for managing SSR, and it holds significant importance for molecular breeding. The MAPK signaling pathway is the foundation for plant responses to pathogens (Meng and Zhang, 2013; Zhang and Zhang, 2022). Activation of the MEKK1-MKK4/MKK5-MPK3/MPK6 pathway has been established as conferring resistance to fungal pathogens in *A. thaliana* (Asai et al., 2002). In rapeseed, *BnMPK3* and *BnMPK6* exhibit heightened responsiveness to *S. sclerotiorum* infection. *BnMPK3*-OE and *BnMPK6*-OE plants showed enhanced resistance to *S. sclerotiorum*, while their RNA interference (RNAi) plants were more susceptible. Importantly, *BnMPK3* and *BnMPK6* confer resistance to rapeseed by positively regulating critical genes of the ET pathway, *BnACS* and *BnEIN3* (Alonso et al., 2003; Lin et al., 2009; Wang et al., 2019a; Wang et al., 2020c). Another MAPK pathway, MEKK1–MKK1/MKK2–MPK4/MPK11, is implicated in defense responses in *A. thaliana* (Zhang and Zhang, 2022). Rapeseeds that overexpress *BnMPK4* have been shown to inhibit *S. sclerotiorum* infection. This overexpression leads to the sustained activation of the expression *PDF1.2* but concurrent inhibition of *PR1* expression (Wang et al., 2009).

WRKY transcription factors constitute one of the largest TF families in higher plants, participating extensively in regulating plant growth, development, plant hormone signal transduction, and various stress responses. They are characterized by a highly conserved WRKY domain, which recognizes and binds to the TTGAC (C/T) W box region in promoters to inhibit or activate downstream gene expression (Rushton et al., 2010; Jiang et al., 2017; Wani et al., 2021). *WRKY33* can be induced by pathogens and regulated by MPK3/MPK6, and WRKY33 can bind to its promoter to regulate expression and induce the synthesis of *A. thaliana* phytoalexin camalexin (Mao et al., 2011). In rapeseed, *BnWRKY33* displays heightened sensitivity to *S. sclerotiorum*, and the enhanced resistance of *BnWRKY33*-OE plants to pathogens is likely linked to the activation of both the SA and JA pathways, as well as redox control (Wang et al., 2014b; Liu et al., 2018a). Conversely, *BnWRKY15*-OE and *BnWRKY28*-OE plants showed increased susceptibility to *S. sclerotiorum*, and the negative regulation of *BnWRKY15* and *BnWRKY28* against pathogen resistance was closely related to the expression of *BnWRKY33* and downstream genes (Liu et al., 2018a; Zhang et al., 2022b). WRKY70 is an activator of the SA pathway and an inhibitor of the JA pathway, *BnWRKY70*-OE plants showed increased susceptibility to *S. sclerotiorum*, while the *Bnwrky70* mutants edited by CRISPR/Cas9 showed stronger resistance to *S. sclerotiorum* (Li et al., 2006b; Sun et al., 2018). Plant mediators (MEDs) interact with RNA polymerase II (RNAP II) and TFs to regulate gene transcription; *AtMED16* regulates the JA/ET pathway and is critical for the recruitment of RNAP II by WRKY33 target genes *PDF1.2* and *ORA59* (Wang et al., 2015). In rapeseed, the expression of *BnMED16* increased following *S. sclerotiorum* infection, BnMED16 interacted with BnMED25 and BnWRKY33, and BnMED25 was closely related to the JA/ET pathway; *BNMED16*-OE plants showed increased resistance to *S. sclerotiorum* (Hu et al., 2021). Heat shock proteins (HSPs) are a class of evolutionarily conserved stress proteins plants produce in response to stress. HSP90, in particular, acts as a molecular chaperone and is essential for the defense response mediated by R proteins (Schulze-Lefert, 2004; Wegele et al., 2004; Sangster and Queitsch, 2005). In rapeseed, 35 *HSP90* genes have been identified, with 6 *BnHSP90* genes exhibiting altered expression induced by *S. sclerotiorum* (Wang et al., 2022a). Since HSP90s interact with many proteins, they may be the key to rapeseed defense response (Tichá et al., 2020).

The SA pathway receptor, NONEXPRESSOR OF PATHOGENESIS-RELATED GENES1 (NPR1), is important for plant systemic acquired resistance (SAR). NPR1 is responsible for promoting the expression of downstream defense genes (Silva et al., 2018; Chen et al., 2021). In rapeseed, 19 NPR1-like genes have been identified, and the expression of *BnNPR1* significantly decreased following *S. sclerotiorum* infection. *BnNPR1*-RNAi plants showed increased sensitivity to *S. sclerotiorum*, resulting in the accumulation of ROS and the suppression of the SA pathway. However, the expression of JA/ET pathway-related genes increased, and this increased sensitivity may be attributed to the early stage role of the SA pathway (Wang et al., 2020b).

GLPs were initially discovered in *Triticum aestivum* and are typically characterized by a cupin domain. Various GLP isoforms exhibit diverse enzymatic activities, including OXO (true germins), superoxide dismutase (SOD), polyphenol oxidase (PPO), and ADP glucose pyrophosphatase/phosphodiesterase (AGPPase) (Lane et al., 1993; Breen and Bellgard, 2010; Barman and Banerjee, 2015). In rapeseed, 14 *GLP* genes have been identified, with *BnGLP3* and *BnGLP12* playing key roles in plant defense and showed SOD activity in the early stages of *S. sclerotiorum* infection (Rietz et al., 2012).

Ca^2+^ is very important in HR and PCD, and they are closely associated with ROS in various plant immune responses (Moeder et al., 2019). Calmodulin-binding activators (CAMTAs) interact with calmodulins (CaMs) to respond to calcium signals, and *CAMTA3* has been identified as a participant in plant defense against pathogens in plants (Koo et al., 2009; Zhang et al., 2014b). Eighteen *CAMTA* genes were identified in the rapeseed genome, and promoter analysis showed that the promoter region of *BnCAMTA* contained many cis-acting elements, and the expression of *BnCAMTA3* was induced by *S. sclerotiorum*. *Atcamta3* mutants of *A. thaliana* showed increased expression of *BAK1* and *JIN1* genes, which are important for plant PTI in response to *S. sclerotiorum*. It appears that CAMTA plays a role in regulating plant immunity to pathogens by negatively regulating PTI and inhibiting the JA pathway (Rahman et al., 2016). Cyclic nucleotide-gated ion channel (CNGC) proteins are known to participate in calcium signal transduction induced by PTI in plants (Tian et al., 2019). In rapeseed, 61 putative *BnCNGC* genes have been classified into five groups (I, II, III, IV-A, and IV-B). Six of these *BnCNGC* genes (four in group I and two in group IV-A) were strongly induced by SA and *S. sclerotiorum*. Susceptible *B. oleracea* displayed increased calcium signaling during *S. sclerotiorum* infection, which might be linked to cell death (Mei et al., 2016; Liu et al., 2021b). However, further studies are required to elucidate the precise function of calcium signal transduction in the *B. napus*–*S. sclerotiorum* system.

Resistance to *S. sclerotiorum* in rapeseed is a complex trait controlled by multiple genes’ quantitative (additive) effects. Recent research has identified several quantitative trait loci (QTLs) associated with *S. sclerotiorum* resistance in rapeseed, with some of these QTLs showing links to flowering time (FT) and yield (Zhao and Meng, 2003; Wei et al., 2014; Li et al., 2015). Doubled-haploid (DH) lines are widely used in QTL mapping studies because they can be consistently identified across different years and experiments (Ding et al., 2021a). Zhao and Meng (2003) used 107 molecular markers to identify six QTLs across various developmental phases. Three QTLs were related to the resistance during the seedling stage. In contrast, the other three were related to resistance during the mature plant stage, and they suggested that different resistance sites may work in different stages or plant organs. The study also observed various epistatic interactions, including dominance × dominance digenic epistasis, in the inheritance of cotyledon resistance (Khan et al., 2020a; Khan et al., 2020b). Wu et al. (2013) mapped 10 QTLs related to stem resistance (SR) during the mature plant stage and 3 QTLs related to leaf resistance (LR) during the seedling stage, *SRC6* on the C6 link explained 29.01% to 32.61% of the phenotypic variation, and the study indicated that *BnaC.IGMT5.a* could be the main candidate gene for this QTL. Yin et al. (2010) used 252 molecular markers and identified a total of 10, 1, and 10 QTLs through mycelial toothpick inoculation (MTI), mycelial plug inoculation (MPI), and infected petal inoculation (IPI), respectively. QTLs on the linkage groups N3, N12, and N17 were detected over 2 years. Zhao et al. (2006) used the petiole inoculation technique to detect 8 QTLs in the HUA population by evaluating days to wilt (DW) and stem lesion length (SLL); four of these QTLs were contributed by resistant parents, and four were contributed by susceptible parents; these results indicated that resistance alleles could be present not only in resistant lines but also in susceptible lines, potentially benefiting breeding efforts. Genome-wide association studies (GWAS) based on linkage disequilibrium (LD) have become a primary tool for identifying gene loci (Wang et al., 2019b). Wei et al. (2016) identified 17 single-nucleotide polymorphisms (SNPs) on A8 and C6 chromosomes by GWAS in different rapeseed accessions with SR to *S. sclerotiorum*. These C6 SNPs were consistent with findings from previous QTL mapping studies. The integration of QTL information is crucial for the molecular breeding of highly resistant rapeseed. Most known R genes typically contain nucleotide-binding site (NBS) and leucine-rich repeat (LRR) domain (Jia et al., 2015). Li et al. (2015) integrated 35 QTLs on 10 chromosomes and identified two conserved SR QTL regions on chromosomes A9 and C6 with several putative NBS-LRR genes clustering at 22.8 and 33.6 Mb, respectively. Furthermore, Wei et al. (2014) found a weak association between rapeseed resistance to *S. sclerotiorum* and FT. They detected common QTL regions, suggesting that early FT and high resistance to *S. sclerotiorum* in rapeseed could benefit future breeding efforts. These findings provide valuable insights into rapeseed resistance to *S. sclerotiorum*.

## Rapeseed transgenic strategy

Rapeseed lacks cultivars with complete resistance to *S. sclerotiorum*; genetic engineering is an effective way to improve rapeseed resistance. Since the degradation of rapeseed cell walls is significant for *S. sclerotiorum* colonization, a promising strategy is to impede pathogen colonization as the first line of plant defense. Overexpressing *PGIPs* typically inhibits pathogen-produced PG and affects pathogen colonization (Federici et al., 2006; Kalunke et al., 2015). Overexpressing *O. sativa OsPGIP2* and *OsPGIP6* in rapeseed has enhanced resistance to *S. sclerotiorum* (Wang et al., 2018; Yin et al., 2022). Chitinase can catalyze the hydrolysis of chitin in fungal cell walls, and co-expressing the chitinase gene *Chit42* from *Trichoderma atroviride* with *PGIP2* from *Phaseolus vulgaris* significantly restricts pathogen growth and delays the disease progression (Cletus et al., 2013; Ziaei et al., 2016). Thaumatin-like proteins (TLPs) belong to the PR5 protein family and play an important role in plant defense (Liu et al., 2010; de Jesús-Pires et al., 2020). Co-expressing chimeric chitinase and *OsTLP* genes have also enhanced rapeseed resistance to *S. sclerotiorum* (Aghazadeh et al., 2016).

Owing to the significant role of OA in *S. sclerotiorum* infection, the overexpression of wheat *OXO* in rapeseed resulted in a remarkable disease reduction rate of up to 90.2% in the sixth-generation lines compared to the parent lines. This breakthrough holds great promise for the development of resistant rapeseed varieties in the future (Dong et al., 2008). However, it is worth noting that while the OXO gene shows substantial potential for enhancing resistance to *S. sclerotiorum*, it is essential to consider that germin and GLPs have been identified as a class of plant allergens. Further research should be conducted to evaluate any potential allergenic effects carefully (Jensen-Jarolim et al., 2002).

Lignin is a polymer composed of p-hydroxyphenyl (H), guaiacyl (G), and syringyl (S) units. It serves to reinforce the mechanical strength of the cell wall and plays a positive role in supporting plant growth and development, and has various functions in mitigating biotic and abiotic stresses (Cesarino, 2019; Dong and Lin, 2021). Ferulate-5-hydroxylase (F5H) is responsible for catalyzing the conversion of G-type units into S-type units within lignin. Knocking out *BnF5H* using CRISPR/Cas9 has been shown to increase the ratio of G-type/S-type units, resulting in enhanced stem strength and improved resistance of stems and leaves to *S. sclerotiorum* (Cao et al., 2022). Strengthening plant tissue mechanically appears to be a viable auxiliary strategy to reduce the impact of SSR. Furthermore, the plant’s surface is covered by a cuticle layer composed of cutin and wax. Research has indicated that changes in cuticular wax composition can influence resistance to *S. sclerotiorum*, in addition to the well-documented role of cutinase in cutin degradation (Wang et al., 2020a; Liu et al., 2021a).

GDSL esterase/lipase has been shown to positively affect pathogen defense responses in *A. thaliana* (Lee et al., 2009). The rapeseed lines overexpressing *AtGDSL1* exhibited reduced JA levels, elevated ROS and SA levels, and increased resistance following *S. sclerotiorum* infection. However, it is worth mentioning that *BnGDSL1*-OE line did not show any changes in resistance; based on the analysis of candidate genes associated with *AtGDSL1* homologs, it was revealed that *BnGLIP1* might be a key gene contributing to rapeseed resistance (Tan et al., 2014; Ding et al., 2020).

Petals play a significant role in *S. sclerotiorum* colonization, and the peptide inflorescence deficient in abscission (IDA) is a key regulator of floral organ abscission (Stø et al., 2015; Shi et al., 2019). An interesting study showed that IDA mutants failed to shed their floral organs properly. When *S. sclerotiorum* was inoculated into the petals of these mutants, although the petals themselves withered and died, the infection did not spread to the leaves as they could not shed normally. This prevented the pathogen from moving between the petals and leaves, thus restricting its transmission (Geng et al., 2022). While most current research focuses on enhancing resistance to *S. sclerotiorum*, typically resulting in disease reduction or delay, it is important to note that ascospores of *S. sclerotiorum* seldom colonize healthy leaves. Therefore, a promising strategy might involve preventing the pathogen’s movement from the heavily colonized petals to healthy leaves or stems, offering an effective means of disease prevention.

Antimicrobial peptides (AMPs) are a class of small cationic peptides known for their wide-ranging antibacterial effects, achieved through mechanisms such as the inhibition of protein transport, cell membrane penetration, and binding to DNA/RNA (Nawrot et al., 2014; Sinha and Shukla, 2019; Bakare et al., 2022). In rapeseed, the identification and validation of the first plant proline-rich antimicrobial peptide (PR-AMP) suggested its potential role in the defense responses of rapeseed (Cao et al., 2015). LjAMP2, a heat-stable antibacterial protein derived from *Leonurus japonicus*, has exhibited the ability to inhibit a range of plant pathogens, and rapeseed that expresses *LjAMP2* showed increased resistance to *S. sclerotiorum* (Yang et al., 2006; Jiang et al., 2013). Similarly, overexpression of *PmAMP1* from *Pinus monticola*, *LTP* from *O. sativa*, and recombinant pathogen-specific antibodies (scFv) in rapeseed also showed increased tolerance to *S. sclerotiorum* (Yajima et al., 2010; Verma et al., 2012; Fan et al., 2013).

Gene editing technology based on the CRISPR/Cas system has emerged as a powerful tool for investigating plant gene functionality, enhancing crop traits, facilitating breeding efforts, and bolstering plant disease resistance (Cardi et al., 2023; Erdoğan et al., 2023; Li et al., 2023). The knockout of susceptibility (S) genes responsible for susceptibility to powdery mildew and stripe rust in wheat has led to enhanced resistance against these two diseases (Wang et al., 2014a; Wang et al., 2022b). SsSSVP1, a small secreted protein from *S. sclerotiorum*, has been found to exert an influence on the subcellular localization of QCR8 (the subunit of the cytochrome b-c1 complex) in *Nicotiana benthamiana*, consequently impairing its biological functions (Lyu et al., 2016). Zhang et al. (2021) identified a homolog of *SsSSVP1* in *Botrytis cinerea* and used CRISPR/Cas9 to reduce the copy number of *BnQCR8*. The mutants showed strong resistance against not only *S. sclerotiorum* but also *B. cinerea*, all without compromising vital agronomic traits in rapeseed. This study provided a novel, effective, and very important strategy for conferring strong resistance in crops against multiple pathogens by editing one gene that encodes a common target of pathogen effectors.

The balance between disease resistance, crop yield, and quality is crucial in agriculture. The discovery of *S. sclerotiorum*-induced promoters is particularly important, and overexpression of resistance genes can enhance plant resistance and cause energy loss (Ding et al., 2021a). The promoter of glycosyl hydrolase 17 gene (*pBnGH17*) in rapeseed was shown to be induced by *S. sclerotiorum*; 5′-deletions and promoter activity analysis showed that a 189-bp region was essential for *S. sclerotiorum* to induce responses, and the promoter *pBnGH17^D7^*, which connects this region to the core promoter region, was induced after *S. sclerotiorum* infection, but it was less active under normal growth conditions (Lin et al., 2022). This provides an important reference for future work. Because of the particularity of transgenic plants, it is necessary to carefully evaluate the biological safety and other aspects in subsequent work.

## Conclusion

In recent years, significant progress in studies of the *B. napus*–*S. sclerotiorum* system has been made, and the transcriptomic and bioinformatics analyses have contributed to the search for potential defense-related genes in rapeseed; genetic engineering and CRISPR/Cas9 also provide new approaches for future research. However, there are still some problems in the current studies. First, although transgenic rapeseed (overexpression/knockout) improved the resistance to *S. sclerotiorum* and delayed disease, no strategy of complete immunity to *S. sclerotiorum* has been reported. Second, the current studies mainly stay in the laboratory stage and lack field experiments, which will be limited to molecular breeding and application. The relationship between yield, quality, and disease resistance should also be balanced. Third, in addition to the search for R genes, the search and development of S genes are equally important; inactivation of the S genes usually induces long and broad-spectrum resistance with little adverse effect on crop growth and yield (Pavan et al., 2010; Zhang et al., 2017; Wang et al., 2022b). In addition, small RNA (sRNA) and host-mediated pathogen gene silencing (cross-kingdom RNAi) have shown great effects in plant defense against pathogens (Wang et al., 2017; Cai et al., 2018), and may be important directions for future studies.

## Author contributions

R-SC: Writing – original draft. J-YW: Writing – review & editing. RS: Writing – review & editing. X-LT: Writing – review & editing.
